# Dental Colleges—Opening

**Published:** 1894-11

**Authors:** 


### Dental Colleges—Opening.

Another year for dental college work has arrived. From information received from a large number of colleges it appears that the number of students in attendance is equal to, if not greater than heretofore. Some colleges have larger classes than ever before, and others about the same number, while some have a few less than heretofore; in the aggregate, however, the number appears to be an increase over any former year, and this, notwithstanding the adverse financial condition of the country, and also in addition to the fact that there is a larger number of colleges in the country than ever before, and also that the requirements for entrance have been increased and the course of study lengthened, and in many instances the curriculum extended. In view of these things how is the increase in numbers to be explained? Is it because the practice of dentistry is more attractive than ever before, and so more inviting, or is entrance into other professions less attractive? It is hardly probable that the latter has much, if any, influence. There is doubtless a better appreciation of the value of dental service and understanding of its capabilities than heretofore. From this, of course, arises a larger demand. The increased requirements and more extensive curriculum necessarily attract the attention of a more highly-educated and better class of students. This we have regarded as an argument in favor of higher requirements, and it would seem, those having charge of our colleges are becoming more fully aware of this fact, and now more readily concur in arrangements calculated to elevate dental education.

During the agitation of these questions the statement was frequently made that extending the time for graduation and raising the requirements for admission would keep back many who would otherwise attend college. This is undoubtedly true; many who formerly were admitted to dental colleges, and some who enter even now, ought to be excluded. While it is true that many are now kept back who would come in under less rigorous requirements, a larger number who are prepared according to the present requirements and are far more appreciative apply, and are admitted, as is fully proven by the largely increased numbers now in attendance over that of former years; and it is not extravagant to assume that if the time was still further extended, the curriculum enlarged, the entrance requirements raised, and the requirements for graduation made more stringent, the numbers would be still further increased and the class of students improved.

This question is now prominently before the educators of our profession, and it is to be hoped that they may clearly see the way open for progress, in all these respects, in the near future.

